# The Anatomage Table: A Promising Alternative in Anatomy Education

**DOI:** 10.7759/cureus.43047

**Published:** 2023-08-06

**Authors:** Eirini-Maria Kavvadia, Ioanna Katsoula, Stavros Angelis, Dimitrios Filippou

**Affiliations:** 1 Anatomy, National and Kapodistrian University of Athens, Athens, GRC

**Keywords:** 3d models, virtual dissection, teaching technique, anatomy, virtual table, anatomage table

## Abstract

Anatomy is one of medical and nursing education’s most prominent and crucial keynotes. For ages, conventional lectures and the analysis of actual human corpses were employed as predominant teaching techniques. However, the sphere of healthcare pedagogy has been greatly altered by the developing passion for technology over the past few years. Anatomage offers a life-size digital representation of the human body, allowing the visualization, manipulation, and virtual dissection of complex anatomical structures, using detailed 3D (three-dimensional) models. Academic institutions utilize Anatomage as a means to enhance and contemporize the acquisition of anatomy knowledge. This systematic review aims to present the educational role of Anatomage in anatomy and whether it can replace the use of cadaveric material in medical education entirely in the future.

A detailed search on PubMed, SCOPUS, Wiley Online Library, and Google Scholar databases was performed. The criteria for the selection were the English language and the year of publication between 2018 and 2023. We rejected publications that were irrelevant to the topic. Before applying the filters, we found 198 publications, from which 24 were finally chosen for the purpose of this review.

The results of this systematic review indicate that most students agree on the beneficial role of Anatomage in the thorough comprehension of anatomical knowledge, and they prefer it over traditional learning methods, such as the use of cadaveric material. Anatomage not only offers a deeper insight into the relations between inner formations, since it is a particularly easy-to-use and pleasant teaching tool, but also contributes to the improvement of learning outcomes in the classroom, which is proved by higher grades in the anatomy course. However, it can be an effective teaching method if it is used in addition to the classic method of cadaver training, rather than being the only educational practice.

Integrating the Anatomage Table (AT) into undergraduate courses is paramount to the comprehensive learning and application of human anatomy in students’ future health careers. Learners who have utilized the table note it to be a beneficial and effective tool in preparing them to enter into the healthcare profession.

## Introduction and background

Anatomy is one of the medical and nursing education’s most prominent and crucial keynotes. A thorough understanding of human anatomy is paramount in the field of medicine, providing vital insights into complex internal structures and serving as a prerequisite for attaining proficiency as a clinician [1].

Historically, textbooks and cadavers have served as the bedrock of teaching and comprehending the structural anatomy of the human body [2]. However, the realm of medical education has been heavily impacted by the developing passion for technology over the past few years. The promotion for incorporating technology into contemporary medical education, specifically utilizing virtual teaching methods such as virtual dissection, has rapidly increased. Doing so allows for a more authentic and detailed visualization of the 3D (three-dimensional) anatomy of virtual cadavers [1,3].

The incorporation of the state-of-the-art Anatomage Table (AT) has greatly elevated the standard of anatomy education within academic establishments. More particularly, California-based company Anatomage Inc. in partnership with the Stanford Clinical Anatomy Department developed Anatomage, a 3D instrument utilized for instructional and diagnostic intentions. In essence, the life-sized virtual human cadaver is displayed on a screen measuring 2.13×0.67 m on the body-size surface, enabling digital dissection. The study of human anatomy is transformed by the AT, which incorporates advanced imaging technologies such as CT scan, X-ray, ultrasound, and MRI, all of which are seamlessly integrated into a user-friendly touchscreen interface [4-6]. Users can dismember the digital body and peel off the skin to display muscles, skeleton, internal organs, nerves, and blood vessels. In addition, they can observe the desired structure in any layer, scale, and size, to understand the relationships between different parts of the body and internal organs. Anatomage offers the opportunity for its users to examine the anatomy of both males and females, as the imagery featured on the platform is originated and licensed from male and female cadavers included in the Visible Human Project (VHP) and Visible Korean Human (VKH). Finally, Anatomage is a versatile educational resource that is not limited to teaching anatomy alone. It is widely used for radiology education, reviewing surgical cases, consulting patients, and conducting research [5,7].

The purpose of this systematic review is to search how Anatomage meliorates the learning procedure of anatomy and whether it can completely supersede the use of cadaveric material in medical education in the foreseeable future.

## Review

Materials and methods

This review is the result of a detailed search in four databases, PubMed, Google Scholar, SCOPUS, and Wiley Online Library, performed with the search terms “Anatomage,” “anatomy education,” and “virtual dissection” using Boolean operators “AND” and “OR.” Possible additional articles were sought by examining the bibliographies of selected articles and journals.

All original studies were included if the publications were in English and were published between 2018 and 2023, while we excluded case reports, case series, letters to the editor, biomechanical studies, animal studies, and expert opinions. We rejected publications that were irrelevant to the topic, and we focused on those in which Anatomage was used for assessing student perception or enhancing learning or was compared to other educational tools used in anatomy education. 

**Figure 1:**
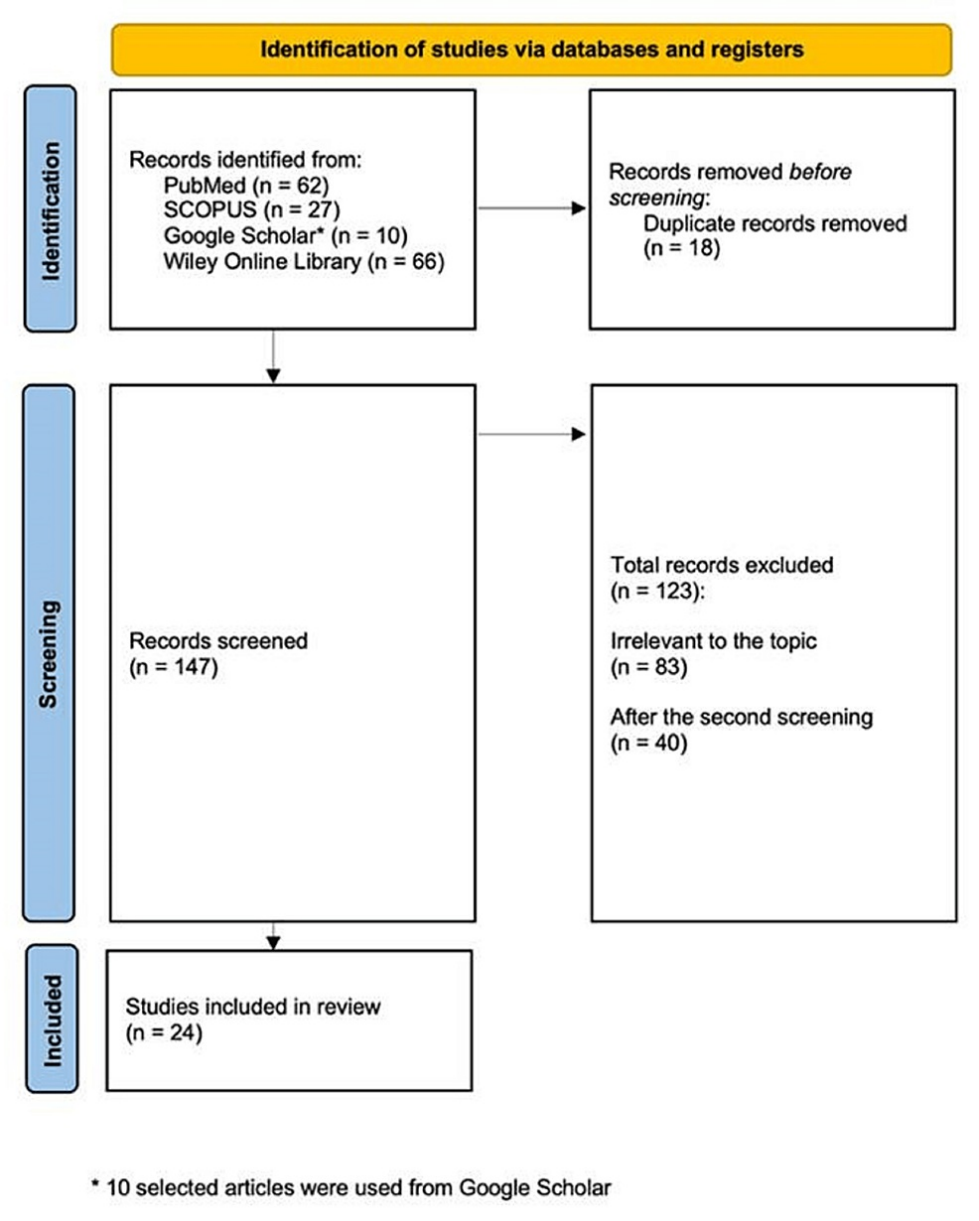
Identification of studies via databases and registers

Before applying the filters, 198 publications were found in PubMed, SCOPUS, and Wiley Online Library. We excluded 43 articles that were not written in English, not accessed in full text, or published before 2018. A total of 10 selected articles were used from Google Scholar. Therefore, 165 articles were identified at first. We further excluded articles irrelevant to the topic, letters to the editor, articles relevant to dentistry and veterinary education, and articles about other dissection tables not including the AT. After the application of the filters, the removal of the duplicates, and the screening process, we ended up with 24 articles that were used in our review. The screening process is explained in the following search flow diagram (PRISMA) (Figure 1). No statistical analysis was used since this was a descriptive study.

Results

Based on this systematic review, it can be concluded that most students believe Anatomage is advantageous in comprehending anatomical knowledge, and they favor it over traditional methods like the practice on cadaveric material. Anatomage not only offers an improved understanding of the connections among internal formations, since it is a particularly easy-to-use and pleasant teaching tool, but also contributes to the improvement of students’ performance in the classroom, which is proved by higher grades in the anatomy course. However, it can be an effective teaching method if it is used in addition to the classic method of cadaver training rather than being the only educational system.

The data extracted are presented in Table 1 and Table 2, showing the key findings as well as the advantages and the complementary role of Anatomage in cadaver-based learning, respectively.

**Table 1:** Key results of Anatomage in anatomy education AT, Anatomage Table; 3D, three-dimensional

	Authors	Title	Year	Key Results
1	Patra A et al. [1]	Integration of innovative educational technologies in anatomy teaching: new normal in anatomy education	2022	1. Possibility to undo any mistakes in virtual dissection. 2. Facilitated teaching Anatomy in medical schools without access to the human body during COVID-19. 3. Contains images of gross and regional anatomy created by digitally tracing real non-chemically treated cadavers.
2	Uruthiralingam U and Rea PM [2]	Augmented and Virtual Reality in Anatomical Education - A Systematic Review	2020	1. Indicates that most articles identified related to both virtual and augmented reality were for the use of new technologies in learning Anatomy. 2. Highlights the recent advances of both augmented reality and virtual reality to implementing the technology into the anatomy course.
3	Onigbinde OA et al. [3]	The place of cadaveric dissection in post-COVID-19 anatomy education	2021	1. Ability to rotate, expand, and minimize organs and tiny structures from numerous angles and planes. 2. No exposure to sharps or accidental cuts. 3. Traditional cadaver dissection related to exposure to harmful chemical foul smells and discomforts caused by embalming chemicals. 4. No emotional engagement in Anatomage as an inanimate object in contrast to physical corpses, which constitute authentic human bodies.
4	Raja BS et al. [4]	Anatomage - the virtual dissection tool and its uses: A narrative review	2022	1. Beneficial as a diagnostic and planning tool in residency programs. 2. Boosts the existing curriculum and helps to counter the challenges with cadaveric dissection as an educational aid.
5	Tenaw B [5]	Teaching gross anatomy: Anatomage table as an innovative line of attack	2020	1. Provides a better understanding of the relationships between internal structures. 2. Allows a 3D presentation of human body parts. 3. Students gain a better spatial perception with the use of haptic tools. 4. Includes both male and female gross anatomy in the software, on a life-size touchscreen table. 5. Allows zooming in and out, manipulating body parts and visualizing them from diverse angles. 6. Promotes independent and collaborative learning. 7. No exposure to sharps or accidental cuts. 8. Traditional cadaver dissection related to exposure to harmful chemical foul smells and discomforts caused by embalming chemicals. 9. In AT embalming products, specially designed space for setting the anatomical table is not necessary
6	Martín JG et al. [6]	Possibilities for the use of Anatomage (the Anatomical Real Body-Size Table) for Teaching and Learning Anatomy with the Students	2018	1. Possibility to dissect in any direction and view structures in three spatial planes. 2. No exposure to sharps or accidental cuts. 3. Traditional cadaver dissection related to exposure to harmful chemical foul smells and discomforts caused by embalming chemicals. 4. In AT embalming products, specially designed space for setting the anatomical table is not necessary
7	Alasmari WA [7]	Medical Students' Feedback on Applying the Virtual Dissection Table (Anatomage) in Learning Anatomy: A Cross-sectional Descriptive Study	2021	1. The majority of students chose to supplement their cadaveric dissection with the 3D Anatomage tool. 2. AT was found advantageous in enhancing students’ anatomy education. 3. Recommends virtual dissection and the AT as additional anatomy learning resources. 4. Efficacy of AT is challenged by errors in labeling and identifying structures and limitations in visualizing anatomical variations
8	Narnaware Y and Neumeier M [8]	Use of a virtual human cadaver to improve knowledge of human anatomy in nursing students: research article	2021	1. Revolutionizes visualizing anatomical structures and performing virtual dissections. 2. Increases the class average in comparison to students taught anatomy without the AT. 3. Recommends virtual dissection and the AT as additional anatomy learning resources. 4. Anatomage offers many advantages as a complementary educational method. 5. Cannot replace cadaveric dissection
9	Bartoletti-Stella A et al. [9]	Three-Dimensional Virtual Anatomy as a New Approach for Medical Student's Learning	2021	1. Beneficial as a supplementary resource in medical education in the assessment of gross anatomy among clinical medical students. 2. Enabled the ongoing study of anatomy during the pandemic. 3. Both male and female gross anatomy are included in the software, on a life-size touchscreen table. 4. Allows zooming in and out, manipulating body parts and visualizing them from diverse angles
10	Boscolo-Berto R et al. [10]	The additional role of virtual to traditional dissection in teaching anatomy: a randomized controlled trial	2021	1. Proven to be beneficial as a supplementary resource in the assessment of gross anatomy among clinical medical students. 2. improves scores of medical students in comparison with those who solely relied on textbooks of topographical anatomy
11	Brucoli M et al. [11]	The potentialities of the Anatomage Table for head and neck pathology: medical education and informed consent	2020	1. Beneficial in the assessment of gross anatomy among clinical medical students as a supplementary resource in medical education. 2. Provides a better understanding of congenital anomalies, anatomical structure variations, and normal and pathological cases. 3. Necessary for the training programs of students and residents and for disease diagnosis and prognosis. 4. Helps in the assessment of students, physicians, and surgeons. 5. Improves the learning outcome of anatomy, radiology, and surgery
12	Said Ahmed MAA. [12]	Use of the Anatomage Virtual Table in Medical Education and as a Diagnostic Tool: An Integrative Review	2023	1. Important educational aid in understanding congenital anomalies, anatomical structure variations, and normal and pathological cases. 2. Necessary for the training programs of students and residents and for disease diagnosis and prognosis. 3. Helps in the assessment of students, physicians, and surgeons. 4. improves the learning outcome of anatomy, radiology, and surgery. 5. Can make undergraduate and postgraduate programs more interesting and valuable
13	Allen MA. et al. [13]	Anatomage Table 6	2019	1. Grants students the chance to independently learn and assess their understanding of anatomy with the inclusion of interactive features. 2. Recommends virtual dissection and the AT as additional anatomy learning resources. 3. Does not provide a multisensory learning experience and diminishes the experiential component that solidifies foundational knowledge gained in the dissection hall. 4. Provides an unrealistic representation of smaller anatomical structures impacting its effectiveness in higher-level education settings. 5. Certain features require prior knowledge of structure origin and perspective
14	Baratz G. et al. [14]	Evaluating the Anatomage Table Compared to Cadaveric Dissection as a Learning Modality for Gross Anatomy	2019	1. Students keen on using technology as a supplementary tool for learning. 2. AT is a more efficient method for retaining information in the short run, albeit only for certain anatomical regions. 3. Anatomage has the potential to achieve similar academic results as those achieved with cadaveric dissection in certain anatomical areas. 4. No discernible variation in scores among students trained using different modalities in studying the musculoskeletal system
15	Bhadoria P. and Modi K. [15]	Virtual dissection – as a new medical teaching tool	2021	1. Provides a better understanding of the relationships between internal structures. 2. Allows a 3D presentation of human body parts. 3. Students gain a better spatial perception with the use of haptic tools. 4. Users can dynamically participate, control, and determine the order, form, and speed of presentation of any information
16	Bin Abdulrahman KA. et al. [16]	Students' Perceptions and Attitudes After Exposure to Three Different Instructional Strategies in Applied Anatomy	2021	1. Users can select and observe any body part in multiple 3D sights. 2. Possibility to undo any mistake in virtual dissection or return to a previous view of the particular structure. 3. Recommends virtual dissection and the AT as additional anatomy learning resources
17	Afsharpour S. et al. [17]	Analysis of immediate student outcomes following a change in gross anatomy laboratory teaching methodology	2018	1. Color-coded images. 2. Possibility to undo any mistakes in virtual dissection
18	Bork F. et al. [18]	The Benefits of an Augmented Reality Magic Mirror System for Integrated Radiology Teaching in Gross Anatomy	2019	1. Recommends virtual dissection and the AT as additional anatomy learning resources. 2. Anatomage improves scores enhancing dissection courses. 3. Both Anatomage and magic mirror are inappropriate for entirely substituting cadaver-based learning
19	Niedermair JF. et al. [19]	On the added benefit of virtual anatomy for dissection-based skills	2023	1. Recommends virtual dissection and the AT as additional anatomy learning resources. 2. Students consider virtual techniques an adjunct but not a replacement for dissection-based anatomy
20	Chytas D. et al. [20]	Do virtual dissection tables add benefit to cadaver-based anatomy education? An evaluation	2022	1. Recommends virtual dissection and the AT as additional anatomy learning resources
21	Owolabi J. et al. [21]	African Medical Educators and Anatomy Teachers' Perceptions and Acceptance of the Anatomage Table as an EdTech and Innovation: A Qualitative Study	2022	1. Anatomy professors agree on the complementary role of Anatomage to cadaver-based learning. 2. Anatomy educators support the continued use of cadaveric dissection as the primary educational method
22	Kar R. et al. [22]	Health Professions Student Perceptions of the Anatomage Virtual Dissection Table and Digital Technology	2020	1. Most students expressed a preference for incorporating 3D Anatomage alongside cadaveric dissection, as an extra resource. 2. Students prefer cadaver dissection over the AT. 3. Anatomage should not be used as a single method to teach and learn human anatomy
23	Sultana Q. et al. [23]	The Impact of Simulation-Based Teaching Module Involving Virtual Dissection on Anatomy Curriculum Delivery	2022	1. Improves scores for students taught with AT as an extra resource in cadaveric dissection
24	https://anatomage.com/table/ [24]	3D Anatomy & Virtual Dissection Platform - Anatomage Table	2023 (Accessed: 01-07-2023)	1. Innovative technological tool with a significant presence in the sectors of medical education and healthcare. 2. Revolutionizes conventional medical diagnosis and treatment-planning methodologies
25	Owolabi J. [25]	Protocol Development for Digisection: Making a Case for Standardizing Educational Technology Use for Digital Dissection and Anatomical Studies	2023	1. Recommends considering finding the balance between traditional teaching practices and the use of educational technology. 2. Standardizing the use of Anatomage in practical anatomy sessions requires the development of protocols, student guides, and continuous improvement of technological skills

**Table 2:** The benefits and the complementary role of Anatomage in the cadaver-based learning AT, Anatomage Table

Grouping Bibliography		
1	Recommending virtual dissection and the AT as additional anatomy learning resources	Alasmari WA [7], Narnaware Y and Neumeier M [8], Bartoletti-Stella A et al. [9], Allen MA et al. [13], Bin Abdulrahman KA et al. [16], Bork F et al. [18], Niedermair JF et al. [19], Chytas D et al. [20], Owolabi J et al. [21], Kar R et al. [22]
2	No exposure to sharps or accidental cuts and harmful chemicals	Onigbinde OA et al. [3], Tenaw B [5], Martín JG et al. [6]
3	Improving scores, enhancing dissection courses	Alasmari WA [7], Narnaware Y and Neumeier M [8], Boscolo-Berto R et al. [10], Brucoli M et al. [11], Said Ahmed MAA [12], Baratz G et al. [14], Bork F et al. [18], Sultana Q et al. [23]
4	The worth of virtual dissection as user-friendly	Onigbinde OA et al. [3], Tenaw B [5], Martín JG et al. [6], Narnaware Y and Neumeier M [8], Bartoletti-Stella A et al. [9], Bhadoria P and Modi K [15], Bin Abdulrahman KA et al. [16], Afsharpour S et al. [17]
5	Facilitates the assessment of students and the lesson	Boscolo-Berto R et al. [10], Brucoli M et al. [11], Said Ahmed MAA [12], Allen MA et al. [13]
6	Beneficial role in diagnosis, prognosis, and treatment	Boscolo-Berto R et al. [10], Brucoli M et al. [11], Said Ahmed MAA [12], anatomage.com [24]

Discussion

The method of visualizing anatomical structures and performing virtual dissections is revolutionized by AT [8]. Using this approach as a supplementary resource to aid in medical education has proven to be beneficial in the assessment of gross anatomy among clinical medical students during their undergraduate training [9-11]. Moreover, Anatomage is an important educational aid in understanding congenital anomalies, anatomical structure variations, and normal and pathological cases, necessary for the training programs of students and residents and for disease diagnosis and prognosis [11,12]. It can also help in the assessment of students, physicians, and surgeons, and it improves the learning outcome of anatomy, radiology, and surgery [11,12]. Introducing Anatomage into the curriculum can make undergraduate and postgraduate programs more interesting and valuable [12]. Furthermore, the inclusion of interactive features, such as the blood flow tool and animated representations of anatomical structures and processes that cannot be replicated in the classroom through traditional teaching methods, grants students the chance to independently learn and assess their understanding of anatomy [13]. Finally, during the pandemic, when dissection practice sessions were suspended, the implementation of Anatomage and other technological methods enabled the ongoing study of anatomy [9]. Boscolo-Berto et al. found that medical students who utilized virtual dissection to study anatomical structures were more than three times as likely to report a positive outcome in the post-gross-dissection test compared to those who solely relied on textbooks of topographical anatomy [10].

As far as students’ performance is concerned, according to Alasmari, the majority of students chose to supplement their cadaveric dissection with the 3D Anatomage tool and found it advantageous in enhancing their anatomy education [7]. According to Baratz et al., students displayed a keenness to utilize technology as a supplementary tool for learning, while the Anatomage group outperformed with a noticeably higher average score on musculoskeletal post-lab quizzes, hinting that the AT is a more efficient method for retaining information in the short run, albeit only for certain anatomical regions [14]. Another study showed that teaching human anatomy using the AT resulted in significant increases in the class average for all three midterm examinations and the final examination in comparison to students taught without the AT [8].

The AT is recognized for providing a better understanding of the relationships between internal structures. It allows a 3D presentation of human body parts and offers haptic tools, with the use of which students can move effortlessly through different layers, study the healthy and pathological forms of various tissues and organs, and gain a better spatial perception [5,15]. Moreover, learners are able to study both male and female gross anatomy through the human cadaver models that are included in the software, on a life-size touchscreen table. They are allowed to zoom in and out, manipulate body parts, and visualize them from diverse angles. In this way, they can identify anatomical structures, the relations, and the connection between them [5,9]. Therefore, they have the ability to rotate, expand, and minimize organs and tiny structures from numerous angles and planes [3]. Furthermore, virtual dissection makes it possible for trainees to cut and make sections of the body in any direction, as well as unite the view of structures at one level with the view in three spatial planes: sagittal and parasagittal, coronal, and transverse [6]. What is more, users can select any body part, such as a particular muscle, nerve, vessel, ligament, or bone, observe it in multiple 3D sights, and undo any mistake in virtual dissection or return to a previous view of the particular structure [16]. Images are color-coded and impossible to destroy by inexperienced dissection, as often happens with the inappropriate preparation and handling of the corpse [17].

Anatomage is characterized by unique interactivity since users can dynamically participate, control, and determine the order, form, and speed of presentation of any information they want to study [15]. Students can use the above feature either individually or in collaboration with others in small groups. Consequently, the AT promotes independent and collaborative learning by developing students' knowledge and skills [5]. Additionally, there are no restrictions or conditions in the use of Anatomage since there is no exposure to sharps or accidental cuts, while in virtual dissection it is not necessary to embalm products or have a space with special characteristics for setting the anatomical table [3,5,6]. On the other hand, traditional cadaver dissection is usually related to exposure to harmful chemical foul smells and discomforts caused by embalming chemicals, such as formalin [3,5,6]. Last but not least, there is no emotional engagement of the learners with the cadaveric models provided by Anatomage since the latter is an inanimate object and virtual reality, in contrast to physical corpses, which constitute authentic human bodies [3].

Despite the advantages of AT in anatomy education that were previously analyzed, this innovative educational tool is more effective when used complementary to traditional teaching methods, without replacing cadaveric dissection. Several studies recommend virtual dissection and the AT as additional anatomy learning resources [7,8,13,16,18-20]. According to the study of Owolabi et al., a high majority of anatomy professors agreed with the previous statement and the complementary role of Anatomage to cadaver-based learning [21]. Although Anatomage is widely regarded as a valuable educational tool by anatomists, educators, and learners, there is no unanimous agreement that it should entirely replace cadaveric dissections. In addition, students also seem to consider contemporary, virtual techniques an adjunct but not a replacement for dissection-based anatomy, stating that the dissection course should keep its position as the key teaching tool in anatomy [19]. In the first large-scale deployment of ATs in two different anatomy courses at UT Health San Antonio, most students expressed a preference for incorporating 3D Anatomage alongside cadaveric dissection, as an extra resource attesting to the usefulness of the virtual 3D Anatomage in anatomy education. However, a significant number of medical students showed their preference for cadaver dissection over the AT, from which we can imply that Anatomage should not be used as a single method to teach and learn human anatomy [22].

Results vary in studies comparing students’ performance when taught with the use of Anatomage or traditional methods. Sultana et al. found that students who were exposed to the virtual dissection table scored comparatively better in post-tests than those exposed to cadaveric dissection [23]. According to Baratz et al., based on the outcomes of the practical exam, it appears that Anatomage has the potential to achieve similar academic results as those achieved with cadaveric dissection in certain anatomical areas. There was no discernible variation in scores among students trained using different modalities in studying the musculoskeletal system [14]. Bork et al. found both Anatomage and the magic mirror virtual tool to offer comparable benefits to dissection courses and greatly enhance them with significantly higher scores for the Anatomage. Nevertheless, both systems were deemed inappropriate for entirely substituting cadaver-based learning [18].

Undoubtedly, Anatomage is an innovative technological tool with a significant presence in the sectors of medical education and healthcare, revolutionizing conventional medical diagnosis and treatment-planning methodologies [24]. However, finding the balance between traditional teaching practices including cadaver dissection and the use of educational technology is a vital consideration [25].

Despite the drawbacks inherent in cadaveric-based learning when compared to the use of Anatomage, the virtual dissection table has to still face the challenge of establishing its role as the primary method in anatomy education. As a technological tool, it lacks the opportunity of providing a multisensory learning experience that can be achieved by working with real human cadavers, and in this way, it diminishes the experiential component that solidifies foundational knowledge gained in the dissection hall [13]. In addition, Anatomage may provide an unrealistic representation of smaller anatomical structures due to a lack of detail and software malfunctions hindering anatomical accuracy and impacting its effectiveness in higher-level education settings [13]. Furthermore, certain features, like the prosection library, require prior knowledge of structure origin and perspective as the images lack orientation labels, making it difficult to decipher anatomical relationships [13]. Errors in labeling and identifying structures as well as limitations in visualizing anatomical variations further challenge the efficacy of Anatomage [7]. According to research, a significant number of anatomy educators support the continued use of cadaveric dissection as the primary educational method recognizing its proven value and reliability over the years [21].

As the literature has already proven, Anatomage might have to offer many advantages as a complementary educational method. However, it cannot replace cadaveric dissection as a subject so complex as anatomy cannot be taught with a sole method [8]. There is still room to investigate the standardization of the use of educational technologies in anatomy education, with Anatomage being the foremost tool in learning anatomy. Standardizing the use of educational technologies including Anatomage in practical anatomy sessions requires the development of protocols, student guides, and continuous improvement of technological skills [25]. Enhancing technical competence and effectively integrating educational technology in anatomy education is crucial for the optimal use of Anatomage in the future.

## Conclusions

The AT offers a futuristic approach to facilitating anatomy education by leveraging technological advancements. Its combination with traditional medical training methods and the use of actual cadavers enhance the effectiveness of virtual dissection instruction. In essence, Anatomage presents a pivotal role in forthcoming education programs as an essential resource for adequately training future healthcare professionals.
